# An exploration of barriers and enablers to the conduct and application of research among complementary and alternative medicine stakeholders in Australia and New Zealand: A qualitative descriptive study

**DOI:** 10.1371/journal.pone.0264221

**Published:** 2022-02-18

**Authors:** Yasamin Veziari, Saravana Kumar, Matthew J. Leach

**Affiliations:** 1 UniSA Allied Health & Human Performance, University of South Australia, North Terrace, Adelaide, South Australia, Australia; 2 Southern Cross University, National Centre for Naturopathic Medicine, East Lismore, New South Wales, Australia; Endeavour College of Natural Health, AUSTRALIA

## Abstract

**Background:**

Most studies examining complementary and alternative medicine (CAM) stakeholder engagement with evidence-based practice have relied on quantitative research methods, which often fail to capture the nuances of this phenomena. Using qualitative methods, this study aimed to explore the experiences of CAM stakeholders regarding the barriers and enablers to the conduct and application of research.

**Methods:**

This research was guided by a qualitative descriptive framework. CAM practitioners and researchers of multiple CAM disciplines from across Australia and New Zealand were invited to share their personal perspectives of the study phenomena. Semi-structured interviews were conducted via Zoom, which were audio-recorded and transcribed verbatim. Rigour strategies were applied to ensure the credibility of results. The transcript was analysed using thematic analysis.

**Results:**

CAM stakeholders identified an array of barriers and enablers to the conduct and application of research within their disciplines. The barriers and enablers that emerged were found to be inter-connected with two similar constructs: capacity and culture. Captured within the construct of capacity were five themes—lack of resources, inadequate governance/leadership, lack of competency, bias directed from outside and within CAM, and lack of time for research. Within the construct of culture were two themes—intrinsic perceptions in CAM, and lack of communication within and outside CAM.

**Conclusions:**

Promoting evidence-based practice and engaging with research in CAM continues to face challenges. This study, for the first time, has highlighted the multitude of interlinked barriers that confront CAM stakeholders when engaging with research. These findings highlight the need for a concerted and targeted approach to tackle these challenges.

## Introduction

Almost five decades have passed since Archie Cochrane challenged conventional health practices, which paved the way for the present-day evidence-based practice (EBP) movement [1]. EBP follows a pathway of steps designed to facilitate the uptake of research evidence in healthcare, including the generation of research evidence; synthesis of the evidence; development of policies that foster EBP uptake; capacity and application of evidence. A multitude of factors have been shown to hamper every step of this pathway [2], from the generation of research [3–6] through to the uptake of research evidence in practice [7–10].

A plethora of perspectives and theories have shed light on the barriers and enablers of EBP [11–14]. Criticisms of the philosophy, methodology, process, and effectiveness of EBP [15–17], together with a conservative disciplinary culture [18], and a perceived lack of value of EBP [19] represent some of the attitudinal barriers to EBP uptake. Structural barriers to EBP include a lack of facilities [20], awareness [8], competence [21], resources [8]. Collectively, these barriers have been shown to negatively impact research generation [22] and evidence utilisation [23]. By contrast, enabling strategies such as research planning, collaboration to develop research, research training [24], fostering an organisational culture, and research support prioritisation [25] have shown some promise in facilitating the uptake of evidence-based practices. Similar barrier issues to EBP have also been reported by research in complementary and alternative medicine [CAM] [26, 27].

CAM represents a range of healthcare practices, technologies, products, and knowledge systems that place particular emphasis on disease prevention, health promotion [28] and treatment [29], but typically sit outside the sphere of the conventional medicine model [30]. As the popularity and use of CAM grows [31–34], there have been increasing calls for these practices to be supported by a growing evidence base [35]; the purpose being to protect CAM consumers from ineffective, unnecessary and/or unsafe interventions [36].

While there have been considerable efforts in addressing the barriers to EBP uptake in conventional healthcare disciplines, research examining EBP in CAM remains in its infancy. This is partly because the evidence-base underpinning many CAM interventions is limited [37–39], resulting in continuing calls from external [40] and internal stakeholders [41] to demonstrate the effectiveness of these therapies. Although building the evidence base of CAM through the conduct of robust research [4, 42] is an important part of the solution, this is unlikely to change practice if the workforce does not have access to appropriate resources and infrastructure, or the capability to translate and implement these research findings into clinical practice [43].

There have been many suggestions on how EBP uptake could be improved in CAM practice [44], yet much of the research to date has relied on quantitative research methods, which often fail to capture the complexities and nuances of this phenomena. Additionally, these studies have been conducted in only a handful of CAM disciplines (i.e., Osteopathy and Chiropractic), and primarily in the United States of America [45–52]. There is currently little known about broader CAM stakeholder engagement with research along the EBP continuum. This research aimed to address this knowledge gap by exploring the opinions of Australian and New Zealand CAM stakeholders on the barriers and enablers to generating and applying research evidence in CAM.

## Methods

### Design

This study used a qualitative descriptive (QD) design in a natural setting to capture the experiences [53], gain personal perspectives, and develop rich descriptive data [54], on the factors impacting the conduct and application of research in CAM [55]. A strength of QD research is the ability to generate findings that remain close to the data, and that present the participant’s reality in everyday language [56]. The consolidated criteria for reporting qualitative research (COREQ) checklist guided the reporting of this study [57].

The broad scope of this research (i.e., the exploration of CAM stakeholder engagement with research across the EBP continuum) closely aligns with the framework for Translating Research into Public Health Action [58]. The framework places research on a spectrum that covers the four domains of *discovery*, *translation*, *dissemination*, *and change*. The premise is that a research idea moves through various stages of transformation until it is adopted in practice or policy. It is acknowledged that the Translating Research into Public Health Action framework is not the “only” lens through which to view this research; however, it does provide as a first step, an organising framework to identify and interpret barriers and enablers to the conduct and application of research in CAM.

### Research question

The aim of this study was to answer the research question, *“What are the opinions of CAM researchers and practitioners regarding the barriers to*, *and enablers of*, *the conduct and application of research in CAM*?*”*

### Recruitment and sampling

The sampling frame for this study was the participant contact list of a recently completed survey, that had examined the barriers to the conduct and application of research. The contact list was generated from a list of survey participants who expressed an interest to participate in this qualitative research via a separate ‘opt-in’ questionnaire. A detailed description of the survey recruitment strategy has been provided elsewhere [59].

This study used non-probability self-selection sampling [60, 61] to ensure participants had experiences relevant to the phenomena, and thus were able to provide information-rich data. As this study aimed to capture the diverse experiences of participants engaged in CAM practice or research [62], the sampling did not focus on a rigid distribution but rather on exploring the phenomena in detail. No standardised guidelines exist for sample size estimates in qualitative studies [62], thus, based on methodological and practical considerations, and previous research [63, 64], the sample size was estimated to be around 20–30 participants [65].

### Participant inclusion/exclusion criteria

Researchers were eligible to participate if they held a post graduate qualification, resided in Australia or New Zealand, had a body of research focused primarily on CAM, had published in their area of expertise, and held an affiliation with a governing organisation. Practitioners had to be residents of Australia or New Zealand, and primarily practice an ingestive or manual CAM therapy, including but not restricted to, Acupuncture, Aromatherapy, Ayurveda, Bowen therapy, Chiropractic, Chinese herbalism, Herbalism, Homeopathy, Kinesiology, Massage therapy, Myotherapy, Naturopathy, nutrition (non-dietetic), Osteopathy, Reflexology and Yoga. Excluded were medical/health researchers, and practitioners who focussed on CAM diagnostic methods (e.g., iridology) or isolated therapeutic techniques (e.g., meditation, Reiki, etc.).

### Interviews

Data were collected using semi-structured interviews as this method affords flexibility on how questions are phrased, ordered, and explained, and which questions should be included or omitted, based on the needs of each participant [66]. The preparation for the interviews followed the interview protocol refinement framework [67]. Development of the interview questions was informed by previous research on this topic [27] and discussions with the research team. These questions were piloted [68] with local CAM stakeholders who were not involved in the main interviews. No changes were made to the interview guide following the pilot test. The interview guide (S1 File) included a broad outline of the topics to be discussed and gave enough time and consideration for the interviewees to elaborate upon their experiences/perceptions.

### Data collection

Interested participants were emailed a participant information sheet, a consent form, and a demographic form prior to the interviews. All interviews were conducted between August and September 2020 over the Zoom videoconferencing platform at a date and time convenient to participants. The interviews were conducted from the lead researcher’s home. Interview times ranged from 20 minutes to 50 minutes. To improve accuracy and aid data interpretation, field notes were taken during and after the interviews. The sole function of the field notes was for cross-checking and recall purposes only and were not included in the data analysis. All interviews were audio-recorded with participant consent, and later transcribed verbatim with identifiers removed. Transcriptions were reviewed for accuracy by the lead researcher and also emailed to all participants for cross-checking, verification, and accuracy [69]. Minor amendments were received from one participant, which were incorporated into the final transcript. By the time 28 participants were interviewed, data saturation had been reached [70].

### Data analysis

NVivo software^™^ [version 10] [71] was utilised to manage data, with each participant assigned a unique identifier to de-identify the data. Data were analysed using thematic analysis, following the six phases of Braun and Clarke: 1) Familiarisation with data (i.e. searching for patterns in the data and translating these into preliminary codes by multiple coders [YV, SK, ML]), 2) systemic coding of data, 3) searching for themes, 4) reviewing themes, 5) defining themes (i.e. aligning themes with sub-themes, and ensuring included quotes covered all interviewee views), and (6) production of the report (i.e. presenting the results utilising appropriate models/frameworks) [72]. Rigour was maintained [73–75] by ensuring credibility (i.e., using three coders [YV, SK, ML]), transferability (i.e., using thick, rich description of the methods), dependability (i.e., triangulation of the data and field notes obtained through observation and member checking) and confirmability of the data (i.e., identifying the limitations and biases of the study), thus ensuring trustworthiness [53, 76].

### Researcher role

By its very nature, qualitative research can foster an imbalance between the researcher and participant [77]. For example, a participant’s perception of the interviewer (i.e., as an educated professional) can influence the information that a participant discloses [78]. Similarly, a researcher can introduce biases that could influence a participant’s perspective. The lead researcher (YV) is a PhD candidate, with formal qualifications in nursing and CAM. She also tutors an undergraduate course in evidence-based practice for health science students. As these educational and experiential biases could not be controlled nor eliminated, several strategies were put in place to manage them. These included undertaking training on the conduct and reporting of qualitative research, adhering to the interview guide and study protocol, reflecting on the research process and findings, and maintaining regular discussions with the research team. Participants were informed of the qualifications of the lead researcher to ensure the relationship between the participant and researcher were neither non-hierarchical, personal nor manipulative.

### Ethics

Ethical approval was obtained from the Human Research Ethics Committee of the University of South Australia (no. 203119). Consent to participate was obtained prior to the conduct of the interviews. Participants were advised of their right to refuse participation or withdraw from the study at any time without consequence. Participants were also given the choice of having the Zoom videoconferencing camera switched on or off during the interviews. Each participant was assigned a unique alpha-numerical identification pseudonym to ensure their data were not identifiable.

## Results

### Participant demographics

Twenty-eight participants from New Zealand (28.5%) and Australia (67.8%) took part in the interviews (Table 1). These participants represented 15 CAM disciplines. Most of the participants were female (78.5%) and aged between 40–59 years (64.2%). More than two-thirds (67.7%) of participants held a higher degree (i.e., Master’s degree or PhD) as their highest qualification. Several participants identified themselves as conducting a combination of even three roles, such as researcher, practitioner, educator, management (42.8%). Most participants had worked in these roles for 11 or more years (60.7%).

**Table 1:** Participant demographics (n = 28).

Characteristic	Frequency
***Country*, *n (%)***	
New Zealand	8 (28.5)
Australia	20 (71.4)
***Gender*, *n (%)***	
Female	22 (78.5)
Male	6 (21.4)
***CAM Disciplines*, *n (%)***	
Acupuncture	1 (3.5)
Aromatherapy	1 (3.5)
Ayurveda	1 (3.5)
Bowen Therapy	2 (7.1)
Chinese medicine	2 (7.1)
Chiropractic	2 (7.1)
Homeopathy	1 (3.5)
Kinesiology	1 (3.5)
Myotherapy	2 (7.1)
Naturopathy	6 (21.4)
Osteopathy	1 (3.5)
Remedial Massage therapy	3 (10.7)
Reflexology	1 (3.5)
Sociology	1 (3.5)
Western Herbal Medicine	3 (10.7)
***Age*, *n (%)***	
Between 20–29 years	0 (0.0)
Between 30–39 years	3 (10.7)
Between 40–49 years	12 (42.8)
Between 50–59 years	6 (21.4)
60 years or more	7 (25.0)
***Highest Qualification*, *n (%)***	
Diploma	1 (3.5)
Advanced diploma	3 (10.7)
Graduate certificate	1 (3.5)
Double degree	1 (3.5)
Post graduate diploma	3 (10.7)
Masters	11 (39.2)
PhD	8 (28.5)
***Professional roles*, *n (%)***	
Researcher only	3 (10.7)
Practitioner only	13 (46.4)
***Combined professional roles*:**	
researcher, practitioner, educator, management	12 (42.8)
***Number of years worked in main professional role*, *n (%)***	
1–5 years	3 (10.7)
6–10 years	8 (28.5)
11–15 years	7 (25.0)
16+years	10 (35.7)

### Understanding the barriers and enablers

**Fig 1:**
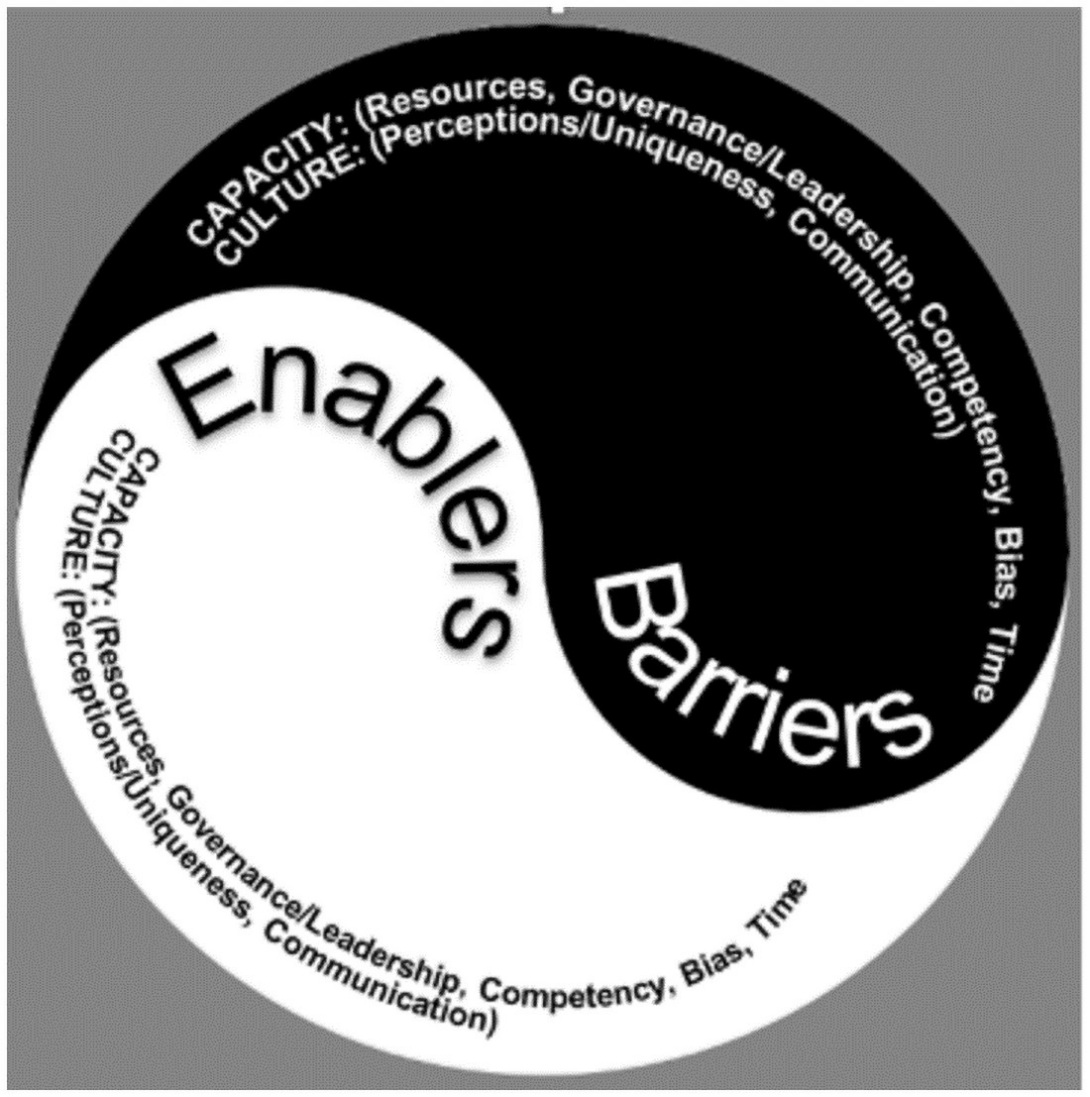
The barriers and enablers to the conduct and application of research by construct and theme.

The analysis of interview transcripts revealed that the barriers and enablers to the conduct and application of research were interconnected and essentially “two sides of the same coin”. Generally, barriers and enablers seemed to focus on one of two broad constructs: capacity and culture. Within these constructs were 8 themes and their 9 sub-themes. Capacity related to issues that were amenable to change with concerted effort and resources. Culture related to issues that influenced the conduct of research but were not readily amenable to change. Fig 1 illustrates how the two constructs, and their integrated themes were categorised into barriers and enablers.

#### 1. Capacity

Within the construct capacity were five themes: resources, governance/leadership, competency, bias, and time.

*Resources*. All participants expressed concerns about inadequate resourcing to undertake research in CAM. The lack of resources was discussed in terms of research support, conduct of research and collaborations.

Research support. Research support was a matter of concern for all participants (n = 28); these encompassed issues such as support systems, a lack of funding, and access to databases. Regarding support systems, some (n = 5) participants felt the inability to garner research support was due to the limited evidence-base in CAM. Academics too felt unsupported to conduct research,

*“I currently now have two supervisors who have no understanding of the area that I actually work in*. *So*, *I’m doing this on my own”*

*(P14)*.

*“You’re not backed by the conventional medical system; you’re not backed by your own field either*. *You’re not backed by anyone”*

*(P22)*.

While there were concerns regarding the lack of support systems for CAM, some enablers to building this support were suggested. Four participants highlighted the need to think outside the box by looking for supports beyond conventional support systems, such as building a network to encourage practitioner engagement with research,

*“I don’t know how you get around it [lack of support systems] but having [something] like PRACI [Practitioner Research and Collaboration Initiative]*, *an international PRACI rather than a national one”*

*(P6)*.

Participants (n = 13) expressed concerns that funding for CAM research was limited. There was a shared sentiment that the “non-patentability” of CAM products could be one reason that also contributes to the lack of funding opportunities for CAM, or even just being associated with CAM deters funding,

*“Good quality public health research often only gets about 25% of the NHMRC research budget*. *So*, *I hate to think what our chances would be from a complementary medicine point of view”*

*(P12)*.

Nine participants reflected on strategies to help overcome barriers to research funding, by exploring funding sources both outside and within CAM. Outside CAM, strategies related to teaming with universities and research centres, non-healthcare commercial entities, and government to attract one-off grants to improve discipline recognition and attract other collaborations. Within CAM, suggestions included, allocating a portion of professional CAM association membership fees to fund research scholarships, conducting student projects to reduce human resource costs, and partnering with industry,

*“…companies that produce products that sell to naturopaths…to donate 1 or 2 cents per product sold*, *that could go into research funding*, *would add up to quite a significant amount of money”*

*(P6)*.

Participants (n = 17) reported that the inability to access databases and full text articles was “*a problem across education*, *practice*, *and research itself” (P6)*. This inaccessibility led to a reliance on, Open Access journals, research from CAM manufacturers, and anecdotal information available on the internet and was due to prohibitive costs, insufficient skills, *“…academic language that you just can’t understand” (P15)*, and being a practitioner,

*“…the published literature is*, *often times it’s not accessible*, *the actual article itself*, *unless you pay or contact the author”*

*(P5)*.

*“I think*, *first of all*, *the accessibility of research is challenging to get to*. *When you’re in practice you don’t necessarily have access to databases that you would do while studying at a university*. *And also*, *once you’ve been out in practice a few years*, *your ability to go and remember how to look for and what to look for in terms of research*, *I think it’s harder to do as well”*

*(P17)*.

Four participants proposed enablers to accessing databases and full text articles, by CAM associations buying membership to journals, involvement with universities, directly emailing researchers, with one suggesting the need for:

*“…a global [CAM] research program that is produced and maybe provided online*, *and colleges purchase that*, *and everybody starts linking through*…”

*(P6)*.

Conduct of research. Barriers to the conduct of research were reported by 8 participants. Some were concerned by the paucity of research conducted in smaller CAM disciplines (e.g., Bowen therapy, Reflexology), and some were concerned about the volume of CAM research conducted by commercial CAM entities,

*“…the “evidence” … that they’re [CAM practitioners] most commonly accessing is funnelled to them from complementary medicine companies that’ve got products to sell*. *And we know the dangers of that*, *in terms of cherry picking of research information and incorrect dissemination of that information and interpretation of it”*

*(P13)*.

Other barriers to the conduct of research related to grant funding (i.e., difficulty accessing and applying for grants), costs (i.e., high costs of conducting large-scale or long-term studies), and resources (i.e., unsuitability of research designs, limited access to instrumentation, lack of research skills, lack of research facilities, lack of connections with mainstream research, lack of established researchers and recruitment difficulties). These multiple barriers were also a deterrent to research engagement. Being a practitioner was “*easier and more financially rewarding” (P17)*. Disregard for conducting research was reflected by another practitioner,

*“I see my evidence with my own eyes every day that I treat patients*. *I don’t need to have a research study show me or tell me that what I do works*. *I know it works”*

*(P9)*.

Suggestions to overcome the barriers to conducting research in CAM included, increasing research engagement (e.g., practitioners volunteering to get involved in research, contacting academics with an interest in CAM) and innovation (e.g., conducting research outside of CAM, such as, workplace surveys, with pharmacists who sell CAM products). Another suggestion was, using case studies to generate foundational evidence:

*“…n = 1*, *where if practitioners are writing up case studies of their own patients*, *or students are doing it at clinic*, *then…do a meta-analysis of all these case studies”*

*(P9)*.

Collaborations. Six participants perceived the CAM industries’ lack of credibility created barriers to collaboration. The inability to collaborate was underpinned by unfamiliarity and culture. Unfamiliarity related to not knowing who to contact for assistance, whereas culture related to the paucity of linkages between the CAM industry and other institutions, interactions between CAM practitioners and universities, and the non-existence of multi-disciplinary research collaborations within CAM,

*“Collaboration is not something we do well in naturopathy historically”*

*(P13)*.

*“If CAM wants to endure in an integrated complementary medicine climate*, *we need to start to develop that evidence-base*. *But to do that*, *we have to develop skills and relationships with research institutions”*

*(P12)*.

Participants (n = 14) proposed building collaborations to address skills, infrastructure, funding, and research support barriers by aligning interests both within and outside CAM. Within CAM, professional associations could collaborate with each other and with the CAM industry; a global research network of PhD graduates also could be created. Outside CAM, suggestions included seeking university employment (to access databases), research involvement (to increase research exposure), engagement with general practitioner clinics (to access patient data, co-author publications and build credibility), and collaborations with mainstream universities (to conduct research projects linking CAM and non-CAM university students). The benefits of student exposure to entities outside CAM were articulated by one participant,

*“Breaking that silo mentality…they [CAM students] will also start thinking about other types of collaboration*, *including research collaboration*…*having something in the undergraduate education to expose to other health disciplines*, *clinical residency programs…so that they get out there and they experience nursing…modern medicine and other disciplines of health care*. *And we build those collaborations that way”*

*(P13)*.

*Governance & leadership*. The lack of involvement in research by professional associations (as representative bodies of CAM professions) was identified as another research capacity issue. Participant attention focussed largely on the leadership roles, and professional obligations of these associations.

Leadership role. Ten participants reflected on the multi-factorial barriers to research leadership by professional associations; these included lack of communication (i.e., between associations), access (i.e., research circulated via associations was usually provided by industry), aspiration (i.e., dearth of research agendas; allowance and provision given to the lowest levels of training, such as certificates), resources (i.e., CAM associations are often run on a voluntary basis, and typically involve multiple competing tasks) and influence (i.e., EBP brought into policy or position statements often becomes a point of contention among the majority of members). Another issue that faced CAM associations was indicated,

*“They [professional associations] have been so stuck in just trying to get over the political attacks*, *and to just foot the agenda forward and have some voice*. *But there hasn’t been much left for doing other things with research”*

*(P9*)

Most participants (n = 19) reported that professional CAM associations could take on a leadership role by promoting research engagement. This engagement could be in the form of promoting the understanding of traditional versus scientific evidence to members, dissemination of current research, and creating research opportunities (e.g., provision of student research competitions, formation of research committees, offering PhD scholarships)–which could be funded through a portion of association membership fees. There was a need for research leaders in CAM, capable of “*straddling” (P9)* both mainstream and CAM, and to “*bridge the research-practice gap” (P1)*. According to one participant, this was a critical step to advancing CAM professions:

*“…*. *they [professional associations] need to understand the importance of research*… *they want regulation*, *and they want integration*, *but…they’re missing their responsibility in that*. *It’s like they don’t get it”*

*(P6)*.

This was not a universal finding however, with one participant placing this responsibility on the individual rather than the associations.

Professional obligation. Participants (n = 6) acknowledged that there was a professional obligation to engage with research. Although, research has now become embedded within the bachelor’s program in CAM, there were limited upskilling opportunities in research for senior practitioners. Seminars and webinars are mainly used for teaching technical skills (e.g., dry needling, hot cupping) and not research focussed,

*“I do feel as a clinician that I have an obligation to stay up to date with information.”*

*(P13)*.

However, research was not necessarily considered a priority for practice or the profession, as one participant articulated,

*“it’s [research] not something that is promoted as being of value and importance for the progression of all the professions within complementary medicine”*

*(P19)*.

Participants (n = 20) felt enablers to professional obligation to engage with research was up to individual and organisational levels. At the individual level, engaging with research was important, especially from a clinical practice context to set an example for one’s peers, and as well as a more formal process from an organisational level,

*“I started lobbying our association in 2016*, *I put a proposal to them about what was needed in terms of research capacity building and on a variety of levels*. *So*, *from that membership skill development level through to organizational infrastructure and looking at partnership arrangements with an academic or some academic institutions*. *I saw that those three elements or levels were really important”*

*(P12)*.

*Competency*. The third theme within the construct of capacity was competency, which comprised of two sub-themes: education, and research skills.

Education. Many participants (n = 16) considered the nature of CAM education in Australia and New Zealand as being a barrier to the conduct and application of research. At the undergraduate level, many CAM programs are delivered in the vocational sector, and a large proportion of these are not informed by national or international education standards. Accordingly, there were variable training standards across many CAM disciplines, with research training often not an explicit requirement of these programs,

*“Our educational pathway is not a formal education*, *so we’re not exposed to the opportunity for research at any point in our early education”*

*(P27)*.

At the post-graduate level, participants reported barriers to accessing training places, which consequently limited opportunities to prepare CAM researchers. Where access to post graduate education did exist, CAM research needed to be modified or become more mainstream or biomedical to be accepted,

*“And the minute I mentioned the word herbal medicine or botanical or holistic to any of the mainstream potential supervisors that I identified*, *they immediately disregarded me as obviously not having something of value there”*

*(P10)*.

Conversely, one participant disagreed about the lack of post-graduate supervisors in CAM but did argue that there was a “*lack of knowledge”* (P 19) about available supervisors.

Several participants (n = 18) suggested a range of enabling strategies to help overcome the educational barriers to building research competency and capacity in CAM. These strategies included embedding research into undergraduate CAM curricula to foster research awareness,

*“What’s really important about the research training is it helps practitioners to understand that pulling together anecdotal evidence is not research”*

*(P12)*.

Extending the academic program from three to four years to create a research-ready workforce, and increasing student exposure to research by inviting researchers to present their work, such as through journal clubs,

*“Well*, *I do think that if there was more exposure to research for undergrads*, *and as they came out into practice*, *that there might be more engagement with even practice-based research*. *If you have 10 practices in a suburb*, *that all gather the same data about something*, *you’ve got a study there”*

*(P27)*.

At the post-graduate level, potential enabling strategies included developing CAM-centric PhD programs to attract CAM practitioners to the university sector and creating a CAM research supervisor directory that could be easily accessed through CAM professional associations.

Research skills. A critical reflection by participants (n = 13) was the wider perception that research in CAM was flawed due to the lack of embedded research skills. Some participants reflected that the training provided in CAM gave little opportunity to learn about research,

*“We weren’t taught to research*… *It’s one of the reasons I haven’t presented a paper on any of my cases or anything because I really don’t know how to do that”*

*(P20)*.

Given the lack of skills, this had a profound effect on implementation as some CAM practitioners reported that they did not have the confidence and cherry-pick data, or skip over parts of, or avoid reading research papers due to the unfamiliarity with statistical information and tests (e.g., p- values),

*“I think my biggest barrier is that I don’t have a university backing or a university degree*. *That’s always been my biggest barrier I think because that limits access to research”*

*(P17)*.

Some participants (n = 5) provided enabling ideas through research literacy training (e.g., mandatory one day workshops, short courses, refresher courses, an online platform of workshops on finding and interacting with research) and postgraduate opportunities,

*“…align myself with people that*… *I’m lucky I’ve got… three other clinics in the CBD [Central Business District] where we’ve got two PhDs and this guy who’s doing his MRes [Masters of Research]”*

*(P18)*.

*Bias*. Captured within the theme of bias, were two sub-themes: attitudes and conformity.

Attitudes. CAM stakeholders (n = 8) perceived there were inherent and systemic negative attitudes towards CAM and CAM research, which to some extent reinforced views that CAM was not evidence-based, as one participant expressed,

*“I find there is heaps of research*, *it is just that the barrier would only come from resistance from certain parts of the medical fraternity in acknowledging or verifying it”*

*(P16)*.

Even when scientifically sound CAM research was produced, participants reported ongoing bias towards CAM research,

*“…sometimes I feel like we’re labelled like we’re not scientific*, *no matter what we’re doing”*

*(P22)*.

Where there was evidence to support the benefits of CAM (e.g., herbal medicines) and no evidence of risk of harm, participants continued to encounter biases in terms of *“the excessive worry about the safety and interaction” (P22)* effects of CAM products. Many participants reported that the difficulties in securing research funding, and limited funding opportunities were a manifestation of these negative attitudes towards CAM research.

While the negative attitudes towards CAM were well acknowledged, strategies to address these barriers were difficult to conceive. One participant felt that *“unless the mechanisms that are creating …exclusions…are addressed” (P14)*, CAM will always be viewed as unscientific.

Conformity. There was a shared view among participants that CAM research had to conform with mainstream medical norms. Some participants (n = 7) felt the need to embrace the dominant epistemology when working in mainstream healthcare systems, and correspondingly, downplay any reference to CAM research. Similarly, some academics felt compelled to embrace mainstream standards and not disclose being part of CAM so that the value of their research was not denigrated, as one participant commented,

*“…I had to normalize my research so that it would fit under the university umbrella*. *…but there was always a little bit of a struggle for me and that I was having to not be completely true to myself in order to get the research done”*

*(P3)*.

Participants believed this perceived need to conform to mainstream medical norms could be mitigated through collaboration with other disciplines, particularly those considered mainstream that value the role of CAM, such as physiotherapy. One participant commented,

*“I think physios are well-placed to help us…if physios were open to including Myo[therapist]s…or Remedial message in their circle*, *then they would find so many that they could then check the inter-rater repetitive stuff and the case studies*, *and they could open up a whole big arm of that*, *then we would collaborate really well”*

*(P2)*.

*Time*. Participants (n = 9) discussed the impact of time and the competing roles they juggle in their careers (i.e., administrator, manager, practitioner) on their capacity to engage with research,

*“I’ve been to conferences where they’ve gone through and covered how to actually try and appraise research*, *but the reality is…On Monday morning*, *you don’t go back and appraise research because you’ve got patients*, *you’ve got a full diary”*

*(P18)*.

Given that many participating CAM practitioners worked in private practice, which generally operate on a fee-for-service model, the lack of funded research time was considered a notable barrier to conducting research. According to one participant:

*“Time is a barrier only because I’m not being paid to do the research*. *So*, *…any research I want to do has to be done in my own time and I’ve got family… I work part time in order that I can try and fit some of my own unpaid work in…”*

*(P25)*.

One participant did offer a suggestion of quarantining set days and times for research activities; however, at a cost (such as employing a research assistant),

*“…having assistance*. *I have a research assistant for my clinical trial*. *Because of that*, *I can allocate a bit less time to my clinical trial*. *It allows me to do more things in other fields such as clinic”*

*(P22*)

#### 2. Culture

Two themes were captured within the construct of culture: perceptions, and communication.

*Perceptions*. Perceptions related to intrinsic perceptions about CAM, and the uniqueness of CAM.

Intrinsic perceptions. Participants (n = 19) reflected on the perceptions of research within CAM. Fundamentally, there was a perceived lack of research culture within CAM, and a particular reliance on traditional evidence. One participant commented on the tension between experiential evidence and the focus on randomised controlled trials,

*“…so*, *I’m studying Ayurveda…The herbs that we use today wouldn’t be used today*, *if there wasn’t 5*,*000 years of experience…there’s a perception… that if somebody talks about evidence*, *they’re only talking about the randomised controlled trial model*, *but I think that there’s a lot to assume when we talk evidence…that evidence includes tradition as well”*

*(P4)*.

While these entrenched views may be difficult to change, some participants questioned the need to change at all. This was shaped by concerns that evidence-based CAM interventions are readily taken up by mainstream health care providers, with CAM being left behind,

*“… as soon as we show the evidence that it works*, *it gets taken by the mainstream anyway”*

*(P9)*.

Several participants (n = 6) highlighted that addressing these barriers would require a concerted effect to recognise and value traditional evidence, as one participant articulated,

*“[It is important] that traditional knowledge doesn’t automatically get thrown out the window when you practice evidence-based medicine or evidence-based health care*. *Actually*, *the opposite is true in that traditional evidence is part of the totality of evidence”*

*(P13)*.

Uniqueness of CAM. Practitioners (n = 14) reflected on the uniqueness of their individualistic approach to patient care, and how this did not typically align with the reductionist approach of mainstream research,

*“Every individual is completely unique and every approach to that person needs to be approached in their own right”*

*(P16)*.

*“…the current methodological processes…considered gold standard…can be quite limiting to the investigation of complex… naturopathic systems that investigate not just a single constituent of a plant*, *but a whole plant with multiple constituents”*

*(P19)*.

While some of this misalignment was due to methodological reasons, others highlighted challenges in measuring aspects of CAM,

*“Because as yoga therapists*, *we might talk about prana*, *moving prana through the body and into the knee and so on and well that’s going to hit a wall*, *isn’t it*? *Scientifically”*

*(P5)*.

This view was not universally shared however, as one participant identified that randomised controlled trials have been successfully used in many CAM disciplines, including Massage therapy, Chiropractic, and Aromatherapy.

Participants (n = 7) reflected on how these barriers could be overcome and suggested developing CAM-centric research designs and methodologies, as well as re-purposing existing data sources to examine the evidence base for CAM,

“*In reality*, *if I do something a thousand times in my clinic and it works*, *I should be able to somehow document that*, *or…there should be another level of research that people can come in… with me and prove that [my] clinical evidence works”*

*(P8)*.

*Communication*. Participants (n = 4) commented on the communication issues prevalent in CAM, and how these impacted engagement with research. These issues included the adoption of a “*silo mentality” (P13)*, and an inability not only to communicate outside CAM but also to communicate with other CAM stakeholders due to differences in viewpoints and mismatched beliefs,

*“There has been a lack of correspondence from academics in the field of naturopathic medicine*, *around the difference between ontology and epistemology”*

*(P14)*.

*“I think a really important barrier between clinicians and researchers that perhaps exists is making sure that clinicians feel they can approach and reach out to researchers in their field with ideas or with issues”*

*(P9)*.

Participants (n = 3) affirmed the need for effective communication between researchers and practitioners to improve research engagement,

“*It’s like… hang on*, *our CAM researchers are there for the same sort of purpose that the practitioners are there*. *Let’s open those conversations up”*

*(P4)*.

## Discussion

This research—the first of its kind in CAM—explored the barriers and enablers to the conduct and application of research from the perspectives of CAM stakeholders across Australia and New Zealand. The findings revealed numerous interlinked barriers, which broadly aligned with the domains of capacity and culture. While barriers to the conduct and application of research have been widely reported in other health disciplines such as allied health [79], medicine [80], and nursing [81], this research identified several barriers unique to CAM. These obstacles included philosophical differences between CAM and biomedicine, and a perceived lack of collaboration, governance, and leadership in CAM.

Despite the plethora of reported barriers to the conduct and application of research in CAM, few enablers were identified. As the reported obstacles are arguably surmountable, this finding may reflect a lack of deliberation or concerted effort among CAM stakeholders to address these barriers. Using established frameworks such as the Translating Research into Public Health Action Framework [58] may provide opportunites to develop enabling strategies targeted at multiple levels (i.e. individual, policy, organisational, environment).

An important finding from this research was that barriers exist across the continuum of evidence, from the generation of evidence, through to access, use and application of evidence. Some of these barriers may relate to cultural norms that determine what constitutes evidence in CAM [82–85]. While traditional evidence, based on centuries of empirical observation [86], largely has been viewed as an extension of clinical experience in CAM [84], this perception may be in a state of flux. There is now ongoing debate about the role and value of traditional evidence in CAM [87], its currency in contemporary practice as a source of valid evidence [83, 88], and its incompatibility with the biomedical view [89, 90].

These cultural norms may be compounded by perceptions that there is minimal need to engage with research and evidence as it is not mandated nor required in CAM [91]. There are also no incentives to promote research in CAM [46, 48]; in fact, given the fee-for-service model that most CAM practitioners operate within [92], engaging with research might be considered a disincentive [93, 94]. Addressing these complex barriers will require a multifaceted approach; one that targets CAM stakeholders to build capacity to engage with research [95], but also supports systems and organisations to transform cultural norms. Such approaches have already been tried, tested, and supported in other health disciplines [95, 96].

Philosophical differences between CAM and biomedical practices were also perceived as barriers to the conduct and application of research in CAM. In CAM, practices are typically planned, delivered, monitored, and viewed through a holistic lens [97–100], which contrasts with the reductionist approach generally favoured in the bio-medical model of care [101]. These philosophical differences [102] may influence how research is valued [100] and viewed by CAM stakeholders [103, 104], producing research which has little commonality between CAM and biomedical practices. One way to address these differences is through collaboration between CAM and biomedicine [105] resulting in shared understanding and co-designed research.

Participants identified the lack of collaboration between CAM and mainstream academic institutions as another important barrier to conducting and applying research in CAM. Fortunately, there have been concerted efforts (albeit limited to the USA) [105–114] aimed at fostering collaboration between CAM and conventional institutions. These studies have consistently shown that collaboration is able to yield mutual benefits in the form of improved research knowledge, skills, competencies, and culture [105]. As CAM and biomedicine are increasingly used together by the population at large [115–118], this is an opportunity for CAM and biomedical professionals to collaborate [119–121] and seek out mutual benefits with much of this responsibility falling on educational institutions.

In addition to building inter-professional relationships, participants commented on the need to improve intra-professional collaboration to unify endeavours. The lack of professional unity in CAM has been widely acknowledged, which can be largely attributed to the diversity of disciplines [122], varying levels of regulation, diverse associations, and agencies [123], and the lack of a unifying governing body. However, overcoming these challenges is not unsurmountable. Indeed, attempts at fostering intra-professional collaboration in medicine and allied health [124, 125] have been shown to improve knowledge and skills development, facilitate shared values, keep practitioners abreast of new innovations [126] and training [127], and assist practitioners in locating expertise [128, 129]. Effective governance and leadership can further facilitate intra-professional collaboration [130] by promoting a shared vision that enables a profession to work towards a common goal [131]–in this case, improving the conduct and application of research in CAM.

### Limitations and strengths

There are some limitations of this study that need to be considered. Despite a comprehensive and broad ranging recruitment strategy (i.e., for the preceding survey [59]), some CAM disciplines were represented by a single participant. There were also more female participants than males, although this is consistent with the gender composition of the Australian and New Zealand CAM workforce [132]. Self-selection sampling also raises the possibility of self-selection bias [61]. At the same time, this study had several strengths. This was the first study to capture the views of different professional groups within CAM (i.e., practitioners, researchers, educators, administrators, managers), as well as diverse disciplines in CAM. To improve the transferability of findings, CAM practitioners were recruited from across different geographical locations. Rigour strategies (e.g., member-checking) were also used to improve the validity of findings.

#### Implications for research

While this research has shed light on the various barriers, and to a lesser extent, enablers to the conduct and application of research in CAM, further research is required to understand this phenomenon further. Such research should focus on exploring each of these barriers in greater depth, as well as understanding the enabling strategies that would work best for individual CAM disciplines and stakeholder groups, including when and why. Future research could explore similar issues that capture a truly multinational perspective, which could bring together findings from several American, Asian, European, and African countries. Findings from this research would contribute toward improving the uptake of evidence-based practices in CAM.

#### Implications for practice

CAM stakeholders are confronted by several barriers to conducting and applying research in CAM yet are exposed to few enabling strategies. With widespread recognition of the need for EBP in CAM, it is important that these barriers are effectively addressed through targeted strategies. Such efforts will require collaboration, commitment, and support both within and beyond CAM to facilitate successful and sustained engagement with research.

## Conclusion

This study has for the first time, highlighted the multitude of interlinked barriers relating to the capacity and culture of EBP that confront CAM stakeholders. Given the numerous barriers identified, it is unlikely that a one-size-fits-all approach will effectively address these obstacles. Therefore, it is recommended that CAM and healthcare stakeholders consider implementing targeted strategies that have been shown to demonstrate positive change. While some of this work has been undertaken, with encouraging findings, there is still more work to be done to ensure these strategies are appropriate and acceptable to diverse CAM disciplines across different jurisdictions.

## Supporting information

S1 File Interview guide. (DOCX)

S1 Checklist Consolidated criteria for reporting qualitative research (COREQ) checklist. (PDF)

## References

[1] Greenhalgh T. Effectiveness and Efficiency: Random Reflections on Health Services. BMJ: British Medical Journal. 2004;328(7438):529-.

[2] Barriers and Bridges to Evidence-Based Clinical Practice. Getting Research Findings Into Practice 2002. p. 115–22.

[3] Ryan J, Daly T. Barriers to innovation and knowledge generation: The challenges of conducting business and social research in an emerging country context. Journal of Innovation & Knowledge. 2018;4.

[4] Karimian Z, Sabbaghian Z, Salehi A, Sedghpour BS. Obstacles to undertaking research and their effect on research output: a survey of faculty members’ views at Shiraz University of Medical Sciences. Eastern Mediterranean health journal = La revue de sante de la Mediterranee orientale = al-Majallah al-sihhiyah li-sharq al-mutawassit. 2012;18(11):1143–50. doi: 10.26719/2012.18.11.1143 23301377

[5] Ataee M, Hesamzadeh A, Kheradmand M. Research barriers from experts’ viewpoints who attended the research workshops of Mazandaran University of Medical Sciences. J Med Life. 2015;8(Spec Iss 4):12–7. 28316700 PMC5319256

[6] Okoduwa SIR, Abe JO, Samuel BI, Chris AO, Oladimeji RA, Idowu OO, et al. Attitudes, Perceptions, and Barriers to Research and Publishing Among Research and Teaching Staff in a Nigerian Research Institute. 2018;3(26).

[7] Straus SE, Tetroe JM, Graham ID. Knowledge translation is the use of knowledge in health care decision making. Journal of Clinical Epidemiology. 2011;64(1):6–10. doi: 10.1016/j.jclinepi.2009.08.016 19926445

[8] Shayan SJ, Kiwanuka F, Nakaye Z. Barriers Associated With Evidence-Based Practice Among Nurses in Low- and Middle-Income Countries: A Systematic Review. 2019;16(1):12–20. 10.1111/wvn.12337 30604471

[9] Zwolsman S, te Pas E, Hooft L, Wieringa-de Waard M, van Dijk N. Barriers to GPs’ use of evidence-based medicine: a systematic review. Br J Gen Pract. 2012;62(600):e511–e21. doi: 10.3399/bjgp12X652382 22781999 PMC3381277

[10] Dizon J, Grimmer K, Louw Q, Machingaidze S, Parker H, Pillen H. Barriers and enablers for the development and implementation of allied health clinical practice guidelines in South African primary healthcare settings: A qualitative study. Health Research Policy and Systems. 2017;15. doi: 10.1186/s12961-017-0243-3 28915890 PMC5603069

[11] Bakken S, Lantigua R, Busacca L, Bigger J. Barriers, Enablers, and Incentives for Research Participation: A Report from the Ambulatory Care Research Network (ACRN). Journal of the American Board of Family Medicine: JABFM. 2009;22:436–45. doi: 10.3122/jabfm.2009.04.090017 19587259 PMC2744643

[12] Mc Goldrick EL, Crawford T, Brown JA, Groom KM, Crowther CA. Identifying the barriers and enablers in the implementation of the New Zealand and Australian Antenatal Corticosteroid Clinical Practice Guidelines. BMC Health Serv Res. 2016;16(1):617. doi: 10.1186/s12913-016-1858-8 27793150 PMC5084422

[13] Jabbour M, Newton AS, Johnson D, Curran JA. Defining barriers and enablers for clinical pathway implementation in complex clinical settings. Implement Sci. 2018;13(1):139-. doi: 10.1186/s13012-018-0832-8 30419942 PMC6233585

[14] Shifaza F, Evans D, Bradley H. Nurses’ Perceptions of Barriers and Facilitators to Implement EBP in the Maldives. Advances in Nursing. 2014;2014.

[15] Greenhalgh T, Snow R, Ryan S, Rees S, Salisbury H. Six ‘biases’ against patients and carers in evidence-based medicine. BMC Med. 2015;13(1):200. 26324223 10.1186/s12916-015-0437-x PMC4556220

[16] Miles A, Loughlin M, Polychronis A. Evidence-based healthcare, clinical knowledge and the rise of personalised medicine INTRODUCTION. Journal of evaluation in clinical practice. 2008;14:621–49. doi: 10.1111/j.1365-2753.2008.01094.x 19018885

[17] Glasziou P, Straus S, Brownlee S, Trevena L, Dans L, Guyatt G, et al. Evidence for underuse of effective medical services around the world. The Lancet. 2017;390(10090):169–77. doi: 10.1016/S0140-6736(16)30946-1 28077232

[18] Donnellan C, Sweetman S, Shelley E. Health professionals’ adherence to stroke clinical guidelines: A review of the literature. Health policy (Amsterdam, Netherlands). 2013;111(3):245–63. doi: 10.1016/j.healthpol.2013.05.002 23727250

[19] Stokke K, Olsen NR, Espehaug B, Nortvedt MW. Evidence based practice beliefs and implementation among nurses: a cross-sectional study. BMC Nursing. 2014;13(1):8. doi: 10.1186/1472-6955-13-8 24661602 PMC3987836

[20] Dale S, Levi C, Ward J, Grimshaw JM, Jammali-Blasi A, D’Este C, et al. Barriers and enablers to implementing clinical treatment protocols for fever, hyperglycaemia, and swallowing dysfunction in the Quality in Acute Stroke Care (QASC) Project—a mixed methods study. Worldviews on evidence-based nursing. 2015;12(1):41–50. doi: 10.1111/wvn.12078 25604606

[21] Leung K, Trevena L, Waters D. Development of a competency framework for evidence-based practice in nursing. Nurse Education Today. 2016;39:189–96. doi: 10.1016/j.nedt.2016.01.026 27006055

[22] Olaussen A, Jennings PA, O’Reilly G, Mitra B, Cameron PA. Barriers to conducting research: A survey of trainees in emergency medicine. Emergency medicine Australasia: EMA. 2017;29(2):204–9. doi: 10.1111/1742-6723.12734 28097829

[23] Baatiema L, Otim ME, Mnatzaganian G, de-Graft Aikins A, Coombes J, Somerset S. Health professionals’ views on the barriers and enablers to evidence-based practice for acute stroke care: a systematic review. Implementation Science. 2017;12(1):74. doi: 10.1186/s13012-017-0599-3 28583164 PMC5460544

[24] Mathieson A, Grande G, Luker K. Strategies, facilitators and barriers to implementation of evidence-based practice in community nursing: a systematic mixed-studies review and qualitative synthesis. Prim Health Care Res Dev. 2019;20:e6–e. doi: 10.1017/S1463423618000488 30068402 PMC6476399

[25] Bach-Mortensen AM, Lange BCL, Montgomery P. Barriers and facilitators to implementing evidence-based interventions among third sector organisations: a systematic review. Implementation Science. 2018;13(1):103. doi: 10.1186/s13012-018-0789-7 30060744 PMC6065156

[26] Leach MJ, Tucker B. Current Understandings of the Research-Practice Gap From the Viewpoint of Complementary Medicine Academics: A Mixed-Method Investigation. Explore (NY). 2017;13(1):53–61. doi: 10.1016/j.explore.2016.10.005 27988241

[27] Veziari Y, Leach M, Kumar S. Barriers to the conduct and application of research in complementary and alternative medicine: A systematic review. BMC Complementary and Alternative Medicine. 2017;17. doi: 10.1186/s12906-017-1660-0 28335766 PMC5364631

[28] Hawk C, Adams J, Hartvigsen J. The Role of CAM in Public Health, Disease Prevention, and Health Promotion. Evidence-Based Complementary and Alternative Medicine. 2015;2015:528487. doi: 10.1155/2015/528487 26819621 PMC4706889

[29] Coulter I, Willis E. The Rise and Rise of Complementary and Alternative Medicine: A Sociological Perspective. The Medical journal of Australia. 2004;180:587–9. doi: 10.5694/j.1326-5377.2004.tb06099.x 15174992

[30] Wieland LS, Manheimer E, Berman BM. Development and classification of an operational definition of complementary and alternative medicine for the Cochrane collaboration. Alternative therapies in health and medicine. 2011;17(2):50–9. 21717826 PMC3196853

[31] Barnes PM, Bloom B, Nahin RL. Complementary and alternative medicine use among adults and children: United States, 2007. National health statistics reports. 2008(12):1–23. 19361005

[32] Conrady D, Bonney A. Patterns of complementary and alternative medicine use and health literacy in general practice patients in urban and regional Australia. Australian Family Physician. 2017;46:315–20. 28472578

[33] Jang A, Kang D-H, Kim DU. Complementary and Alternative Medicine Use and Its Association with Emotional Status and Quality of Life in Patients with a Solid Tumor: A Cross-Sectional Study. The Journal of Alternative and Complementary Medicine. 2017;23(5):362–9. doi: 10.1089/acm.2016.0289 28453297 PMC5446597

[34] Chrystal K, Allan S, Forgeson G, Isaacs R. The use of complementary/alternative medicine by cancer patients in a New Zealand regional cancer treatment centre. The New Zealand medical journal. 2003;116(1168):U296. 12601420

[35] Kinsel JF, Straus SE. Complementary and alternative therapeutics: rigorous research is needed to support claims. Annual review of pharmacology and toxicology. 2003;43:463–84. doi: 10.1146/annurev.pharmtox.43.100901.135757 12540748

[36] Ekor M. The growing use of herbal medicines: issues relating to adverse reactions and challenges in monitoring safety. Front Pharmacol. 2014;4:177-. 10.3389/fphar.2013.00177 PMC3887317 24454289

[37] Power M, Hopayian K. Exposing the evidence gap for complementary and alternative medicine to be integrated into science-based medicine. J R Soc Med. 2011;104(4):155–61. doi: 10.1258/jrsm.2011.100271 21502214 PMC3078611

[38] Tabish SA. Complementary and Alternative Healthcare: Is it Evidence-based? Int J Health Sci (Qassim). 2008;2(1):V–IX. 21475465 PMC3068720

[39] MacArtney JI, Wahlberg A. The Problem of Complementary and Alternative Medicine Use Today: Eyes Half Closed? Qualitative Health Research. 2014;24(1):114–23. doi: 10.1177/1049732313518977 24406483

[40] Fischer FH, Lewith G, Witt CM, Linde K, von Ammon K, Cardini F, et al. High prevalence but limited evidence in complementary and alternative medicine: guidelines for future research. BMC complementary and alternative medicine. 2014;14:46-. doi: 10.1186/1472-6882-14-46 24499316 PMC3931324

[41] Steel A, McEwen B. The need for higher degrees by research for complementary medicine practitioners. Australian Journal of Herbal Medicine. 2014;26:136+.

[42] Crowe S, Turner S, Utley M, Fulop NJ. Improving the production of applied health research findings: insights from a qualitative study of operational research. Implementation Science. 2017;12(1):112. doi: 10.1186/s13012-017-0643-3 28886709 PMC5591553

[43] Curtis K, Fry M, Shaban RZ, Considine J. Translating research findings to clinical nursing practice. J Clin Nurs. 2017;26(5–6):862–72. doi: 10.1111/jocn.13586 27649522 PMC5396371

[44] Leach MJ, Canaway R, Hunter J. Evidence based practice in traditional & complementary medicine: An agenda for policy, practice, education and research. Complementary therapies in clinical practice. 2018;31:38–46. doi: 10.1016/j.ctcp.2018.01.011 29705478

[45] Leach MJ, Gillham D. Evaluation of the Evidence-Based practice Attitude and utilization SurvEy for complementary and alternative medicine practitioners. Journal of Evaluation in Clinical Practice. 2008;14(5):792–8. doi: 10.1111/j.1365-2753.2008.01046.x 19018912

[46] Schneider MJ, Evans R, Haas M, Leach M, Hawk C, Long C, et al. US chiropractors’ attitudes, skills and use of evidence-based practice: A cross-sectional national survey. Chiropr Man Therap. 2015;23:16-. doi: 10.1186/s12998-015-0060-0 25949800 PMC4422535

[47] Leach MJ, Gillham D. Are complementary medicine practitioners implementing evidence based practice? Complementary Therapies in Medicine. 2011;19(3):128–36. doi: 10.1016/j.ctim.2011.04.002 21641517

[48] Bussières AE, Terhorst L, Leach M, Stuber K, Evans R, Schneider MJ. Self-reported attitudes, skills and use of evidence-based practice among Canadian doctors of chiropractic: a national survey. J Can Chiropr Assoc. 2015;59(4):332–48. 26816412 PMC4711333

[49] Roecker CB, Long CR, Vining RD, Lawrence DJ. Attitudes toward evidence-based clinical practice among doctors of chiropractic with diplomate-level training in orthopedics. Chiropr Man Therap. 2013;21(1):43. doi: 10.1186/2045-709X-21-43 24314309 PMC4029280

[50] Tilburt JC, Curlin FA, Kaptchuk TJ, Clarridge B, Bolcic-Jankovic D, Emanuel EJ, et al. Alternative medicine research in clinical practice: a US national survey. Arch Intern Med. 2009;169(7):670–7. doi: 10.1001/archinternmed.2009.49 19364996 PMC2804465

[51] Walker BF, Stomski NJ, Hebert JJ, French SD. A survey of Australian chiropractors’ attitudes and beliefs about evidence-based practice and their use of research literature and clinical practice guidelines. Chiropr Man Therap. 2013;21(1):44. doi: 10.1186/2045-709X-21-44 24345082 PMC3878410

[52] Sundberg T, Leach M, Thomson O, Austin P, Fryer G, Adams J. Attitudes, skills and use of evidence-based practice among UK Osteopaths: a national cross-sectional survey. Advances in Integrative Medicine. 2019;6:S44–S5. 10.1186/s12891-018-2354-6 PMC6286591 30526551

[53] Creswell JW. Research design: Qualitative & quantitative approaches. Thousand Oaks, CA, US: Sage Publications, Inc; 1994. xix, 228–xix, p.

[54] Neergaard MA, Olesen F, Andersen RS, Sondergaard J. Qualitative description–the poor cousin of health research? BMC Med Res Methodol. 2009;9(1):52. 19607668 10.1186/1471-2288-9-52 PMC2717117

[55] Polit D, Beck C. Nursing Research:- Generating and Assessing Evidence for Nursing Practice. China: Wolters Kluwer| Lippincott Williams & Wilkins; 2012.

[56] Sandelowski M. Whatever happened to qualitative description? 2000;23(4):334–40. 10.1002/1098-240x(200008)23:4<334::aid-nur9>3.0.co;2-g 10940958

[57] Tong A, Sainsbury P, Craig J. Consolidated criteria for reporting qualitative research (COREQ): a 32-item checklist for interviews and focus groups. International Journal for Quality in Health Care. 2007;19(6):349–57. doi: 10.1093/intqhc/mzm042 17872937

[58] Brownson RC, Kreuter MW, Arrington BA, True WR. Translating scientific discoveries into public health action: how can schools of public health move us forward? Public health reports (Washington, DC: 1974). 2006;121(1):97–103. doi: 10.1177/003335490612100118 16416704 PMC1497798

[59] Veziari Y, Kumar S, Leach M. Barriers to the conduct and application of research among complementary and alternative medicine professions in Australia and New Zealand: A cross-sectional survey. Complementary Therapies in Medicine. 2021;60:102752. doi: 10.1016/j.ctim.2021.102752 34126172

[60] Khazaal Y, van Singer M, Chatton A, Achab S, Zullino D, Rothen S, et al. Does self-selection affect samples’ representativeness in online surveys? An investigation in online video game research. Journal of medical Internet research. 2014;16(7):e164–e. doi: 10.2196/jmir.2759 25001007 PMC4115258

[61] Eysenbach G, Wyatt J. Using the Internet for Surveys and Health Research. Journal of medical Internet research. 2002;4:E13. doi: 10.2196/jmir.4.2.e13 12554560 PMC1761932

[62] Malterud K, Siersma VD, Guassora AD. Sample Size in Qualitative Interview Studies: Guided by Information Power. Qualitative health research. 2016;26(13):1753–60. doi: 10.1177/1049732315617444 26613970

[63] Mason M. Sample Size and Saturation in PhD Studies Using Qualitative Interviews. Forum Qualitative Sozialforschung / Forum: Qualitative Social Research. 2010;11.

[64] Francis JJ, Johnston M, Robertson C, Glidewell L, Entwistle V, Eccles MP, et al. What is an adequate sample size? Operationalising data saturation for theory-based interview studies. Psychology & health. 2010;25(10):1229–45. doi: 10.1080/08870440903194015 20204937

[65] Morse J. Determining Sample Size. Qualitative Health Research. 2000;10:3–5.

[66] Robson C, McCartan K. Real World Research, 4th Edition2017.

[67] Castillo-Montoya M. Preparing for interview research: The interview protocol refinement framework. 2016;21:811–31. doi: 10.1016/j.circir.2016.05.008 27422801

[68] McGrath C, Palmgren PJ, Liljedahl M. Twelve tips for conducting qualitative research interviews. Medical Teacher. 2019;41(9):1002–6. doi: 10.1080/0142159X.2018.1497149 30261797

[69] Birt L, Scott S, Cavers D, Campbell C, Walter F. Member Checking: A Tool to Enhance Trustworthiness or Merely a Nod to Validation? Qualitative health research. 2016;26(13):1802–11. doi: 10.1177/1049732316654870 27340178

[70] Glaser B, Strauss A. The discovery of grounded theory: Strategies for qualitative research. New York: Aldine Publishing Company; 1967.

[71] NUD*IST Vivo (N-Vivo). 10 ed. Melbourne: Qualitative Solutions and Research Pty. Ltd.; 1999.

[72] Braun V, Clarke V. Using thematic analysis in psychology. Qualitative Research in Psychology. 2006;3(2):77–101.

[73] Hofseth LJ. Getting rigorous with scientific rigor. Carcinogenesis. 2018;39(1):21–5. doi: 10.1093/carcin/bgx085 28968787 PMC5862244

[74] Forero R, Nahidi S, De Costa J, Mohsin M, Fitzgerald G, Gibson N, et al. Application of four-dimension criteria to assess rigour of qualitative research in emergency medicine. BMC Health Serv Res. 2018;18(1):120. doi: 10.1186/s12913-018-2915-2 29454350 PMC5816375

[75] Koch T. Establishing rigour in qualitative research: the decision trail. Journal of Advanced Nursing. 2006;53(1):91–100. doi: 10.1111/j.1365-2648.2006.03681.x 16422698

[76] Patton M. Qualitative Research And Evaluation Methods. http://lst-iiepiiep-unescoorg/cgi-bin/wwwi32exe/[in=epidoc1in]/?t2000=018602/(100). 2002;3.

[77] Råheim M, Magnussen LH, Sekse RJT, Lunde Å, Jacobsen T, Blystad A. Researcher–researched relationship in qualitative research: Shifts in positions and researcher vulnerability. International Journal of Qualitative Studies on Health and Well-being. 2016;11(1):30996. doi: 10.3402/qhw.v11.30996 27307132 PMC4910304

[78] Richards H, Emslie C. The ‘doctor’ or the ‘girl from the University’? Considering the influence of professional roles on qualitative interviewing. Family practice. 2000;17:71–5. doi: 10.1093/fampra/17.1.71 10673494

[79] Matus J, Wenke R, Hughes I, Mickan S. Evaluation of the research capacity and culture of allied health professionals in a large regional public health service. J Multidiscip Healthc. 2019;12:83–96. doi: 10.2147/JMDH.S178696 30666124 PMC6336030

[80] Friesen EL, Comino EJ. Research culture and capacity in community health services: results of a structured survey of staff. Australian journal of primary health. 2017;23(2):123–31. doi: 10.1071/PY15131 27531587

[81] Luckson M, Duncan F, Rajai A, Haigh C. Exploring the research culture of nurses and allied health professionals (AHPs) in a research-focused and a non-research-focused healthcare organisation in the UK. J Clin Nurs. 2018;27(7–8):e1462–e76. doi: 10.1111/jocn.14264 29322683

[82] Hao P, Jiang F, Cheng J, Ma L, Zhang Y, Zhao Y. Traditional Chinese Medicine for Cardiovascular Disease: Evidence and Potential Mechanisms. Journal of the American College of Cardiology. 2017;69(24):2952–66. doi: 10.1016/j.jacc.2017.04.041 28619197

[83] Wiese M. Does ‘traditional’ evidence have a place in contemporary complementary and alternative medicine practice? A case for the value of such evidence. Focus on Alternative and Complementary Therapies. 2016;21:143–6.

[84] Wardle J. Respecting science, respecting tradition: Evidence-based care in the integrative medicine professions. Australian Journal of Herbal Medicine. 2015;27:47–55.

[85] World Health Organzation. WHO Traditional Medicine Strategy 2014–2023. Geneva; 2013.

[86] Patwardhan B, Mashelkar RA. Traditional medicine-inspired approaches to drug discovery: can Ayurveda show the way forward? Drug discovery today. 2009;14(15–16):804–11. doi: 10.1016/j.drudis.2009.05.009 19477288

[87] Lemonnier N, Zhou G-B, Prasher B, Mukerji M, Chen Z, Brahmachari SK, et al. Traditional Knowledge-based Medicine: A Review of History, Principles, and Relevance in the Present Context of P4 Systems Medicine. Progress in Preventive Medicine. 2017;2(7). doi: 10.1097/pp9.0000000000000010 29457147 PMC5812272

[88] Abbott R. Documenting Traditional Medical Knowledge 2014.

[89] Milstein M. The case against alternative medicine. Can Vet J. 2000;41(10):769–72. 11062833 PMC1476381

[90] Shahvisi A. No Understanding, No Consent: The Case Against Alternative Medicine. Bioethics. 2016;30(2):69–76. doi: 10.1111/bioe.12228 26806449

[91] Hunt K, Ernst E. Evidence-based practice in British complementary and alternative medicine: double standards? Journal of Health Services Research & Policy. 2009;14(4):219–23. doi: 10.1258/jhsrp.2009.009009 19770119

[92] Australian Bureau of Statistics. Health Care Services, Chiropractic and Osteopathic Services. Canberra: The Bureau; 2011. Report No.: 8570.0.

[93] Ikegami N. Fee-for-service payment—an evil practice that must be stamped out? Int J Health Policy Manag. 2015;4(2):57–9. doi: 10.15171/ijhpm.2015.26 25674568 PMC4322626

[94] Magill MK. Time to Do the Right Thing: End Fee-for-Service for Primary Care. Ann Fam Med. 2016;14(5):400–1. doi: 10.1370/afm.1977 27621155 PMC5394371

[95] Goodman MS, Sanders Thompson VL. The science of stakeholder engagement in research: classification, implementation, and evaluation. Translational behavioral medicine. 2017;7(3):486–91. doi: 10.1007/s13142-017-0495-z 28397159 PMC5645283

[96] Sharplin G, Adelson P, Kennedy K, Williams N, Hewlett R, Wood J, et al. Establishing and Sustaining a Culture of Evidence-Based Practice: An Evaluation of Barriers and Facilitators to Implementing the Best Practice Spotlight Organization Program in the Australian Healthcare Context. Healthcare (Basel, Switzerland). 2019;7(4):142. doi: 10.3390/healthcare7040142 31726668 PMC6956050

[97] Jagtenberg T, Evans S, Grant A, Howden I, Lewis M, Singer J. Evidence-based medicine and naturopathy. J Altern Complement Med. 2006;12(3):323–8. doi: 10.1089/acm.2006.12.323 16646733

[98] Marian F, Widmer M, Herren S, Dönges A, Busato A. Physicians’ philosophy of care: a comparison of complementary and conventional medicine. Forsch Komplementmed. 2006;13(2):70–7. doi: 10.1159/000090735 16645286

[99] O’Leary D. Medicine’s metaphysical morass: How confusion about dualism threatens public health. Synthese. 2020. 10.1007/s11229-020-02869-9 PMC7512202 32989333

[100] Stub T, Foss N, Liodden I. "Placebo effect is probably what we refer to as patient healing power": A qualitative pilot study examining how Norwegian complementary therapists reflect on their practice. BMC complementary and alternative medicine. 2017;17(1):262-. doi: 10.1186/s12906-017-1770-8 28499371 PMC5429571

[101] Barrett B, Marchand L, Scheder J, Appelbaum D, Plane MB, Blustein J, et al. What complementary and alternative medicine practitioners say about health and health care. Ann Fam Med. 2004;2(3):253–9. doi: 10.1370/afm.81 15209203 PMC1466673

[102] Mazzotta C. Biomedical approaches to care and their influence on point of care nurses: a scoping review. Journal of Nursing Education and Practice. 2016;6.

[103] Tonelli MR, Callahan TC. Why alternative medicine cannot be evidence-based. Acad Med. 2001;76(12):1213–20. doi: 10.1097/00001888-200112000-00011 11739043

[104] Verhoef MJ, Vanderheyden LC, Dryden T, Mallory D, Ware MA. Evaluating complementary and alternative medicine interventions: in search of appropriate patient-centered outcome measures. BMC Complementary and Alternative Medicine. 2006;6(1):38. doi: 10.1186/1472-6882-6-38 17118197 PMC1661594

[105] Cramer GD, Guiltinan J, Maiers M, Laird S, Goertz C, Furner SE, et al. Benefits, Challenges, and Culture Change Related to Collaborations Between Complementary and Alternative Medicine and Traditional Research-Intensive Institutions. Medical Science Educator. 2015;25(1):27–34.

[106] Allen ES, Connelly EN, Morris CD, Elmer PJ, Zwickey H. A train the trainer model for integrating evidence-based medicine into a complementary and alternative medicine training program. Explore (NY). 2011;7(2):88–93. doi: 10.1016/j.explore.2010.12.001 21397869 PMC6448140

[107] Evans R, Delagran L, Maiers M, Kreitzer MJ, Sierpina V. Advancing evidence informed practice through faculty development: the Northwestern Health Sciences University model. Explore (NY). 2011;7(4):265–8. doi: 10.1016/j.explore.2011.04.014 21724163 PMC3408000

[108] Haas M, Leo M, Peterson D, Lefebvre R, Vavrek D. Evaluation of the effects of an evidence-based practice curriculum on knowledge, attitudes, and self-assessed skills and behaviors in chiropractic students. J Manipulative Physiol Ther. 2012;35(9):701–9. doi: 10.1016/j.jmpt.2012.10.014 23206965 PMC3515209

[109] Laird S, George J, Sanford SM, Coon S. Development, implementation, and outcomes of an initiative to integrate evidence-based medicine into an osteopathic curriculum. J Am Osteopath Assoc. 2010;110(10):593–601. 21068224 PMC3142697

[110] Long CR, Ackerman DL, Hammerschlag R, Delagran L, Peterson DH, Berlin M, et al. Faculty development initiatives to advance research literacy and evidence-based practice at CAM academic institutions. J Altern Complement Med. 2014;20(7):563–70. doi: 10.1089/acm.2013.0385 24936915 PMC4086219

[111] McCarty RL, Fenn R, Gaster B, Weber W, Guiltinan J. Building bridges: qualitative assessment of a clinical faculty exchange between a naturopathic and an allopathic medical training program. Explore (NY). 2011;7(4):249–53. doi: 10.1016/j.explore.2011.04.003 21724159 PMC3129540

[112] Sullivan B, Furner S, Cramer G. Development of a Student Mentored Research Program between Complementary and Alternative Medicine and Traditional, Research Intensive Universities to Foster Evidence-based Practitioners and Clinician-researchers. EXPLORE: The Journal of Science and Healing. 2013;9:323.

[113] Wayne P, Buring J, Davis R, Andrews S, St. John M, Kerr C, et al. Increasing research capacity at the New England School of Acupuncture through faculty and student research training initiatives. Alternative therapies in health and medicine. 2008;14:52–8. 18383990

[114] Zwickey H, Schiffke H, Fleishman S, Haas M, Cruser dA, LeFebvre R, et al. Teaching evidence-based medicine at complementary and alternative medicine institutions: strategies, competencies, and evaluation. J Altern Complement Med. 2014;20(12):925–31. doi: 10.1089/acm.2014.0087 25380144 PMC4270143

[115] Stoneman P, Sturgis P, Allum N, Sibley E. Incommensurable Worldviews? Is Public Use of Complementary and Alternative Medicines Incompatible with Support for Science and Conventional Medicine? PloS one. 2013;8:e53174. doi: 10.1371/journal.pone.0053174 23382836 PMC3559728

[116] Avila C, Grace S, Bradbury J. How do patients integrate complementary medicine with mainstream healthcare? A survey of patients’ perspectives. Complementary Therapies in Medicine. 2020;49:102317. doi: 10.1016/j.ctim.2020.102317 32147079

[117] Shim JM. The Relationship Between the Use of Complementary and Alternative Medicine and the Use of Biomedical Services: Evidence From East Asian Medical Systems. Asia-Pacific journal of public health. 2016;28(1):51–60. doi: 10.1177/1010539515613411 26512027

[118] Zank S, Hanazaki N. The coexistence of traditional medicine and biomedicine: A study with local health experts in two Brazilian regions. PLOS ONE. 2017;12(4):e0174731. doi: 10.1371/journal.pone.0174731 28414735 PMC5393556

[119] Sullivan T. Collaboration: a heathcare imperative. New York: McGraw-Hill; 1998.

[120] Cant S. Medical Pluralism, Mainstream Marginality or Subaltern Therapeutics? Globalisation and the Integration of ‘Asian’ Medicines and Biomedicine in the UK. Society and Culture in South Asia. 2020;6(1):31–51.

[121] Wade C, Chao M, Kronenberg F, Cushman L, Kalmuss D. Medical pluralism among American women: results of a national survey. J Womens Health (Larchmt). 2008;17(5):829–40. doi: 10.1089/jwh.2007.0579 18537484 PMC2942786

[122] Gaboury I, April KT, Verhoef M. A qualitative study on the term CAM: is there a need to reinvent the wheel? BMC complementary and alternative medicine. 2012;12:131-. doi: 10.1186/1472-6882-12-131 22909051 PMC3462712

[123] Mills SY. Regulation in complementary and alternative medicine. BMJ (Clinical research ed). 2001;322(7279):158–60. doi: 10.1136/bmj.322.7279.158 11159577 PMC1119419

[124] Bridges DR, Davidson RA, Odegard PS, Maki IV, Tomkowiak J. Interprofessional collaboration: three best practice models of interprofessional education. Medical education online. 2011;16: doi: 10.3402/meo.v16i0.6035 21519399 PMC3081249

[125] Nancarrow SA, Booth A, Ariss S, Smith T, Enderby P, Roots A. Ten principles of good interdisciplinary team work. Human resources for health. 2013;11:19. doi: 10.1186/1478-4491-11-19 23663329 PMC3662612

[126] Ray CE Jr., Sirota DJ. Collaboration between Industry and Physicians-An Essential Alliance. Semin Intervent Radiol. 2013;30(4):331–2. doi: 10.1055/s-0033-1359725 24436558 PMC3835441

[127] Ross JS, Keyhani S, Korenstein D. Appropriateness of collaborations between industry and the medical profession: physicians’ perceptions. Am J Med. 2009;122(10):955–60. doi: 10.1016/j.amjmed.2009.04.013 19786162 PMC3020980

[128] Dean KS, editor Strategies and Benefits of Fostering Intra-Organizational Collaboration 2010.

[129] Herman PM, Coulter ID. Complementary and Alternative Medicine: Professions or Modalities? Policy Implications for Coverage, Licensure, Scope of Practice, Institutional Privileges, and Research: RAND Corporation; 2015.

[130] Kaiser R, Hogan R, Craig B. Leadership and the Fate of Organizations. The American psychologist. 2008;63:96–110. doi: 10.1037/0003-066X.63.2.96 18284278

[131] Chhotray S, Sivertsson O, Tell J. The Roles of Leadership, Vision, and Empowerment in Born Global Companies. Journal of International Entrepreneurship. 2018;16(1):38–57.

[132] Leach MJ. Profile of the complementary and alternative medicine workforce across Australia, New Zealand, Canada, United States and United Kingdom. Complement Ther Med. 2013;21(4):364–78. doi: 10.1016/j.ctim.2013.04.004 23876568

