# Supplementary material for: An exploration of barriers and enablers to the conduct and application of research among complementary and alternative medicine stakeholders in Australia and New Zealand: A qualitative descriptive study
Source: PLoS One. 2022 Feb 18;17(2):e0264221. doi: 10.1371/journal.pone.0264221 (PMC8856519; doi:10.1371/journal.pone.0264221)
Supplement: S1 File — (DOCX) [file pone.0264221.s001.docx]

| **List of topic headings and prompts** |
| --- |
| **1) Engagement with research**   - How do you engage with research?   **2) Barriers Encountered**   - As researchers, we often find that there are barriers to doing research. Could you share some of the barriers that you may have encountered when engaging with research?   i. If you did, how did you come across this barrier?  **3) Strategies/Enablers**   - Did you overcome the barrier?   i. If you did, tell me what happened, what strategies did you use?  ii. Did this work for you?  iii. If it did not work for you? Why did it not work for you?   - Have you come across any supports (industry, association, governing body) to facilitate/enable a research culture?   i. If yes, what were they? 4**) Additional prompts**   - large-scale research - long-term research - collaborations - support (institutional) - access to evidence - knowledge and skills - professional obligation - funding   **5) Recommendations**   - How do you think some of these barriers can be addressed?   i. Have you tried any of these strategies?  ii. If so, what has worked? What has not worked?   - You have identified a number of barriers and enablers and recommendations; in moving forward, what do you think the situation would look like if those recommendations were in place? |
